# Detection of β-Lactams and Chloramphenicol Residues in Raw Milk—Development and Application of an HPLC-DAD Method in Comparison with Microbial Inhibition Assays

**DOI:** 10.3390/foods7060082

**Published:** 2018-06-01

**Authors:** Eftychia Karageorgou, Sofia Christoforidou, Maria Ioannidou, Evdoxios Psomas, Georgios Samouris

**Affiliations:** Laboratory of Food Hygiene and Technology, Veterinary Research Institute, Hellenic Agricultural Organization-DEMETER, Campus of Thermi, 57001 Thessaloniki, Greece; efi_karag@yahoo.gr (E.K.); xristof_sof@yahoo.gr (S.C.); ioannidou@vri.gr (M.I.); psomas@vri.gr (E.P.)

**Keywords:** antibiotic residues, raw milk, HPLC, MSPD, microbial inhibition assays

## Abstract

The present study was carried out to assess the detection sensitivity of four microbial inhibition assays (MIAs) in comparison with the results obtained by the High Performance Liquid Chromatography with Diode-Array Detection (HPLC-DAD) method for antibiotics of the β-lactam group and chloramphenicol in fortified raw milk samples. MIAs presented fairly good results when detecting β-lactams, whereas none were able to detect chloramphenicol at or above the permissible limits. HPLC analysis revealed high recoveries of examined compounds, whereas all detection limits observed were lower than their respective maximum residue limits (MRL) values. The extraction and clean-up procedure of antibiotics was performed by a modified matrix solid phase dispersion procedure using a mixture of Plexa by Agilent and QuEChERS as a sorbent. The HPLC method developed was validated, determining the accuracy, precision, linearity, decision limit, and detection capability. Both methods were used to monitor raw milk samples of several cows and sheep, obtained from producers in different regions of Greece, for the presence of examined antibiotic residues. Results obtained showed that MIAs could be used effectively and routinely to detect antibiotic residues in several milk types. However, in some cases, spoilage of milk samples revealed that the kits’ sensitivity could be strongly affected, whereas this fact does not affect the effectiveness of HPLC-DAD analysis.

## 1. Introduction

Antibiotics are widely used in veterinary practice for the treatment of microbial infections or diseases and as dietary supplements. The excessive and inconsiderate use of antibiotics may lead to the occurrence of drug residues in milk, bearing a risk to human health, since they can cause allergic reactions in hypersensitive individuals, or they may result in drug-resistant bacteria. Thus, the antibiotic residues’ analysis is important to guarantee food safety [1].

Regulatory authorities such as the European Union (EU) and the US Food and Drug Administration, in order to ensure food safety, have enacted not only strict Maximum Residue Limits (MRLs) that allow only trace amounts of antimicrobial residues in milk for human consumption, but also requirements concerning the performance of analytical methods and the interpretation of the results [1].

Beta-lactam antibiotics are among the most frequently used antimicrobial agents, and include penicillin derivatives, cephalosporins, monobactams, carbapenems, and β-lactamase inhibitors. Cephalosporins and penicillins have similar chemical structures, both bearing β-lactamic rings [2].

Chloramphenicol is a synthetic antibiotic with a phenylpropanoid structure with similar broad spectrum. Its use in animals is illegal in most countries [3].

The methods being used for the detection and/or determination of antibacterial reagents in milk are divided into two major groups. The first group consists of the screening methods (MIAs, rapid test kits, enzyme-linked immunosorbent assay, surface plasmon resonance technology biosensor) [4], which are economical, easy to use, and have high sample through-put, but are characterized by low sensitivity and low specificity [5,6]. The second group comprises confirmatory methods, mainly including separation techniques such as liquid chromatography [4], which assess the target analytes’ quantification, have high specificity and provide structure elucidation when it is coupled with mass spectrometers [5,6].

In the field of separation techniques, LC (Liquid Chromatography) is the most frequently applied one, usually followed by either diode array UltraViolet (UV) detection [2,7,8,9,10,11,12,13,14] or mass spectrometry [15,16,17,18,19,20,21].

Prior to chromatographic analysis, a sample pretreatment technique should be applied for the effective isolation of antibiotics from complex milk matrix. Sample preparation techniques commonly used include extraction of target analytes with a variety of organic solvents followed by SPE (Solid Phase Extraction) [10,18], solid phase microextraction [14] and liquid–liquid extraction [11,16,19,20]. During recent years, modern extraction techniques are being used for the same purpose, including solid phase extraction using molecularly imprinted polymers as the sorbent material [9,12,17,21], matrix solid-phase dispersion (MSPD), and dispersive SPE applying the quick, easy, cheap, effective, rugged, and safe (QuEChERS) methodology [2,7,8,13,15].

The EU reference method for the antibiotic residues’ determination in raw and heat-treated milk is the International Dairy Federation (IDF) microbial inhibitor assay dependent on the use of *Bacillus stearothermophilus*, a microorganism which is selected because of its superior sensitivity to antibiotics. However, due to technical difficulties that may occur carrying out the reference method, microbial inhibitor assays based on the IDF method are used as routine methods for detecting the antibacterial substances in milk [22]. These microbial tests are fast, economical, easy to perform, and need no sample preparation prior to analysis. They rely on the germination and growth of *Bacillus stearothermophilus* spores, which initiate an acidification process that causes the change of a pH indicator from purple to yellow. When residues are present, the spores will not germinate and the pH indicator will remain unchanged [22]. However, it should be noted that several factors such as the natural inhibitors in colostrums, high or low pH values, increased somatic cell or bacterial counts, and milk compositional changes can affect the sensitivity of the microbial inhibition assays [23].

There are no microbial inhibitor tests that can detect all substances at the MRLs set by the EU Regulations. The majority of the methods are designed for the group of β-lactams, as they are the most common veterinary drugs in the therapy of cows in many countries [24].

Between 2006 and 2012, seven notifications by the European Rapid Alert System for Food and Feed (RASFF) specifically reported the detection of antibiotics including β-lactams and chloramphenicol in milk and milk products [25].

Several studies on milk safety and purity by the application of screening methods on the antibiotic residues detection have been reported [26].

The scope of the present research was to compare the results of raw milk analysis for the detection of antibiotic residues by microbial inhibitor tests available in Greek market, versus the results of the analysis by a more precise and sophisticated method using HPLC-DAD technique. The selection of β-lactams was based on the frequent use they present in veterinary medicine in Greece, especially in intramammary administration, whereas chloramphenicol was chosen due to its severe impacts on public health due to exposure. To the best of our knowledge, this is the first attempt of such a comparative study to be conducted in Greece.

## 2. Materials and Methods

### 2.1. Instrumentation

#### 2.1.1. HPLC-DAD Instrumentation

The HPLC system used for the chromatographic determination of antibiotic residues in milk was the Perkin Elmer Series 200, equipped with a Photo-Diode Array (PDA) detector and 100 μL loop (Perkin-Elmer, Shelton, CT, USA). Degassing of the mobile phase was achieved by vacuum degasser Perkin Elmer Series 200 directly in the solvent reservoirs. A Perfectsil ODS-2, 5 μm, 250 × 4 mm analytical column, purchased from MZ-Analysentechnik, Mainz, Germany was used for the separation of examined analytes.

A glass vacuum filtration apparatus obtained from Alltech Associates was employed for the filtration of ammonium acetate, using cellulose nitrate 0.45 μm membrane filters (Sartorius Stedim Biotech GmbH, Gottingen, Germany). A Vortex Genie 2, (Bohemia, NY, USA) and an ultrasonic bath AM-9 Aquasonic Cleaners (Sherwood, AR, USA) were used for the pretreatment of milk samples. All evaporations were performed with a 16-port evaporator model from Barkey GmbH & Co. KG (Leopoldshöhe, Germany).

Two SPE products were investigated with regard to their efficiency for the isolation of β-lactams in milk: Plexa (60 mg/3 mL), Agilent Technologies Inc. (Lake Forest, CA, USA) and Oasis-HLB (200 mg/6 mL) by Waters (Milford, MA, USA). Two mL dispersive SPE tubes QuEChERS containing 150 mg magnesium sulfate, 50 mg PSA (primary, secondary amines) and 50 mg C_18_EC also purchased by Agilent Technologies, were used in MSPD mode.

#### 2.1.2. Microbial Inhibitor Tests and Instruments Used

In order to evaluate the microbial inhibition assays available in the Greek market for the simultaneous and rapid detection of several antibiotic residues in raw cow’s milk, four test kits were obtained and compared: (a) ECLIPSE 3G (ZEU IMMUNOTEC, Spain), (b) Charm Blue Yellow II (Charm Sciences Inc., Lawrence, MA, USA), (c) BRT MRL Screening test (AIM, München, Bavaria, Germany) and (d) Delvotest SP-NT (DSM Food Specialties, Delft, The Netherlands). For ethical reasons, the kits will from now on be referred to as “A”, “B”, “C” and “D”, respectively.

The preparation of standard solutions and spiking of milk samples at the appropriate concentrations was achieved by the use of calibrated pipettes Eppendorf Research plus 0.5-10 μL, 10–100 μL and 20–200 μL. After inoculation, microplates were sealed and incubated at a circulating water bath Memmert (MEMMERT GmbH + Co. KG, Schwabach, Germany) at 64–65 °C for 2.5–3.5 h, depending on the manufacturers’ instructions. Results were evaluated either visually by observing the wells’ colors in comparison to reference colors, or photometrically by the use of ELISA reader TECAN infinite F50 (Grödig, Austria) and special scanners Epson Perfection V600 Photo and Epson Perfection V30 (Epson Europe B.V., Amsterdam, The Netherlands), kindly provided from kits’ manufacturers.

### 2.2. Reagents and Materials

Amoxicillin trihydrate (AMO), ampicillin trihydrate (AMP), oxacillin sodium salt monohydrate (OXA), cloxacillin sodium salt monohydrate (CLO), ceftiofur (CFU), cefalonium hydrate (CFU) and chloramphenicol (CAP) were purchased from Sigma-Aldrich (Steinheim, Germany). Cefazolin CRS (CFZ) and cefapirin sodium CRS (CFP) used were European Pharmacopoeia References. HPLC grade methanol, acetonitrile and water used, whereas acetone and ammonium acetate were of analytical grade and obtained from Sigma-Aldrich (Steinheim, Germany).

Raw cow’s milk was obtained from individual small milk-producing facilities in the regional rural zone of Thessaloniki, Greece. The milk was obtained in the middle of milk production from healthy cows not being treated with antibiotics and was firstly analyzed by the HPLC method so as to be confirmed free of antibiotic residues and sanitizers that could interfere with the kits’ sensitivity. The initial milk sample was prepared in aliquots of 50 mL and was stored at −20 °C until the day of analysis.

### 2.3. Standard Solution Preparation

For the chromatographic analysis, aqueous stock standard solutions of each antibiotic were prepared at a concentration of 100 ng μL^−1^. Ceftiofur was prepared in methanol in the same concentration. Cephalosporins stock solutions were stable for six months, whereas penicillins and chloramphenicol were stable for one month when stored at 4 °C. Working aqueous standards were freshly prepared daily by further dilution at various concentrations. Aliquot of 100 μL were injected onto the column and quantitative analysis was based on peak area measurements.

For the analysis by microbial inhibition assays, all stock standard solutions were freshly prepared on the day of analysis at a concentration of 100 ng μL^−1^. Chloramphenicol and β-lactams, except for ceftiofur, were dissolved in sterile demineralized water, while ceftiofur was dissolved in pure methanol according to the manufacturer’s instructions. Subsequent dilutions were prepared in sterile demineralized water and cow’s milk to yield appropriate working standard solutions at various concentrations ranging from 50 to 150% MRL. During the analysis, all solutions were maintained on sterile ice. For chloramphenicol, a substance whose presence in food is prohibited in the EU, higher concentrations than MRL were chosen to evaluate the kits’ sensitivity.

### 2.4. Chromatography

Target analytes were separated by gradient elution using A: CH_3_COONH_4_ 0.05 M and B: ACN. The initial volume ratio was 90:10 (*v*/*v*) and was kept isocratic for 7 min. Over the next 3 min, this ratio was changed to 80:20 (*v*/*v*) and was kept isocratic for 10 min. Over the next 5 min, the ratio became 70:30 (*v*/*v*) and was also kept isocratic for 15 min. During the last 5 min of analysis, the mobile phase returns to its initial composition. Flow rate at 0.9 mL/min provides approximately inlet pressure 2500 psi. The analytical column was operated at ambient temperature and PDA detector was set at 240 and 265 nm. The evaluation software was Total Chrom V6.2.0.0.1 with LC instrument control Perkin Elmer.

### 2.5. Sample Preparation Prior to HPLC-DAD Analysis

The reversible adsorption capabilities of different commercially available sorbents for isolation of antibiotics were evaluated, and the optimum sorbent was applied to milk on dispersive extraction by QuEChERS in MSPD format, where the extraction was enhanced ultrasonically. The MSPD procedure was applied using Plexa sorbent. The SPE sorbent material was preconditioned by flushing 2 mL of methanol and 2 mL of water. Then it was emptied into a glass beaker, where 500 μg of milk and 500 μL of standard solution of examined antibiotics, in the case of spiked samples, with half the quantity of a QuEChERS tube—that is, 125 mg—added. In the modified MSPD method used herein, homogenization was enhanced by sonication for 10 min in an ultrasound bath. Afterwards, the sample was transferred into an empty cartridge reservoir and compressed. At this step the sorbent was dried by vacuum. Subsequently, interference was removed by washing the sorbent bed twice with 5 mL water (1% acetone), and finally the analytes of interest were eluted with 2 mL methanol. The samples were filtrated with PVDF Durapore syringe filters (13 mm × 0.45 μm) Millex Millipore Corporation (Bedford, MA, USA) prior to evaporation until dryness under nitrogen stream, and the residues were dissolved in 500 μL of water. Aliquots of 100 μL of the resulting samples were injected into the HPLC system. In the case of milk samples, the same procedure was followed by adding 500 μL of distilled water instead of 500 μL standard solution. 

### 2.6. Analysis by Means of Microbial Inhibition Assays

Four microbial inhibitor tests were used for the qualitative analysis of antibiotic residues in cow’s milk. The kits were stored at 2 °C and left 1 h at room temperature before use. Each commercial kit was used following the manufacturers’ instructions. Milk samples for spiking were brought to the refrigerator to defrost one day before the analysis took place. The assay was performed in 96-well breakable microplates with *Geobacillus stearothermophilus var. calidolactis* spores in agar. Analyses were done in triplicate for every antibiotic concentration tested. For the interpretation of the results, in each assay one positive and one negative control were used in quadruplicate. All controls were freshly prepared on the day of analysis according to the manufacturers’ instructions, and the results of all tests were interpreted both visually by color comparison and photometrically using specific scanners provided by the kits’ manufacturers. The detection limits of the test kits are presented in Table 1.

## 3. Results and Discussion

### 3.1. Chromatography

The multistep gradient elution program yielded optimum separation of the target antibiotics within 45 min. Quantitation of target analytes was performed at the wavelength of optimum absorbance for each analyte, as follows: AMO, CLO and OXA at 240 nm, CFZ, CFP, CFU, CFN and CAP at 265 nm.

### 3.2. Sample Preparation Prior to HPLC-DAD Analysis

The optimum SPE sorbent material was Plexa, which, during the optimization experiments in standard solutions, proved to gain high absolute recoveries for all examined antibiotics (65–99%). Different MSPD approaches were tested in order to optimize the method in terms of recovery rates and efficient matrix clean-up. Results are shown in Table 2.

**Figure 1:**
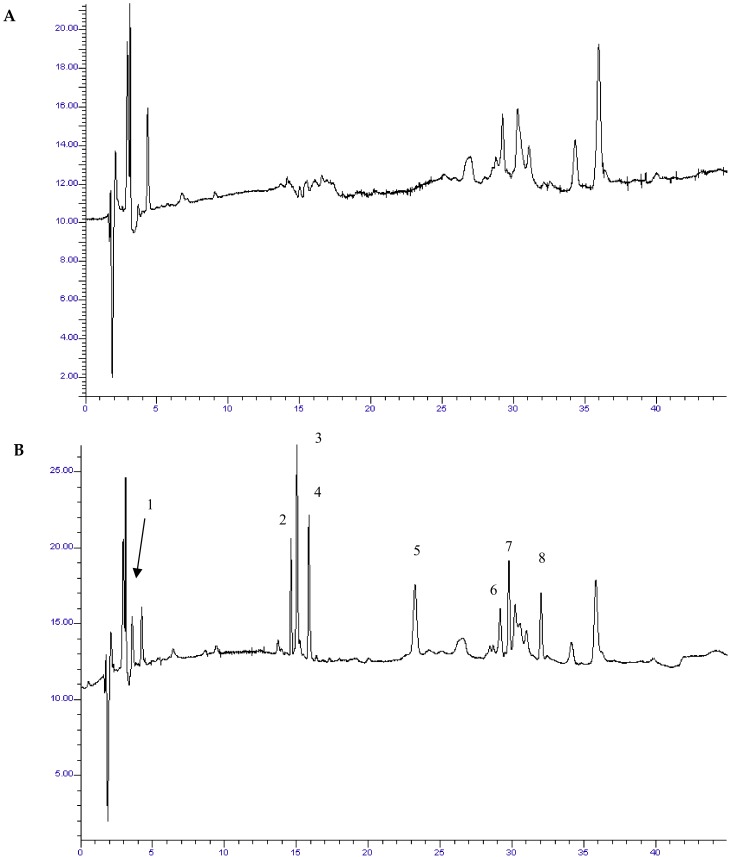

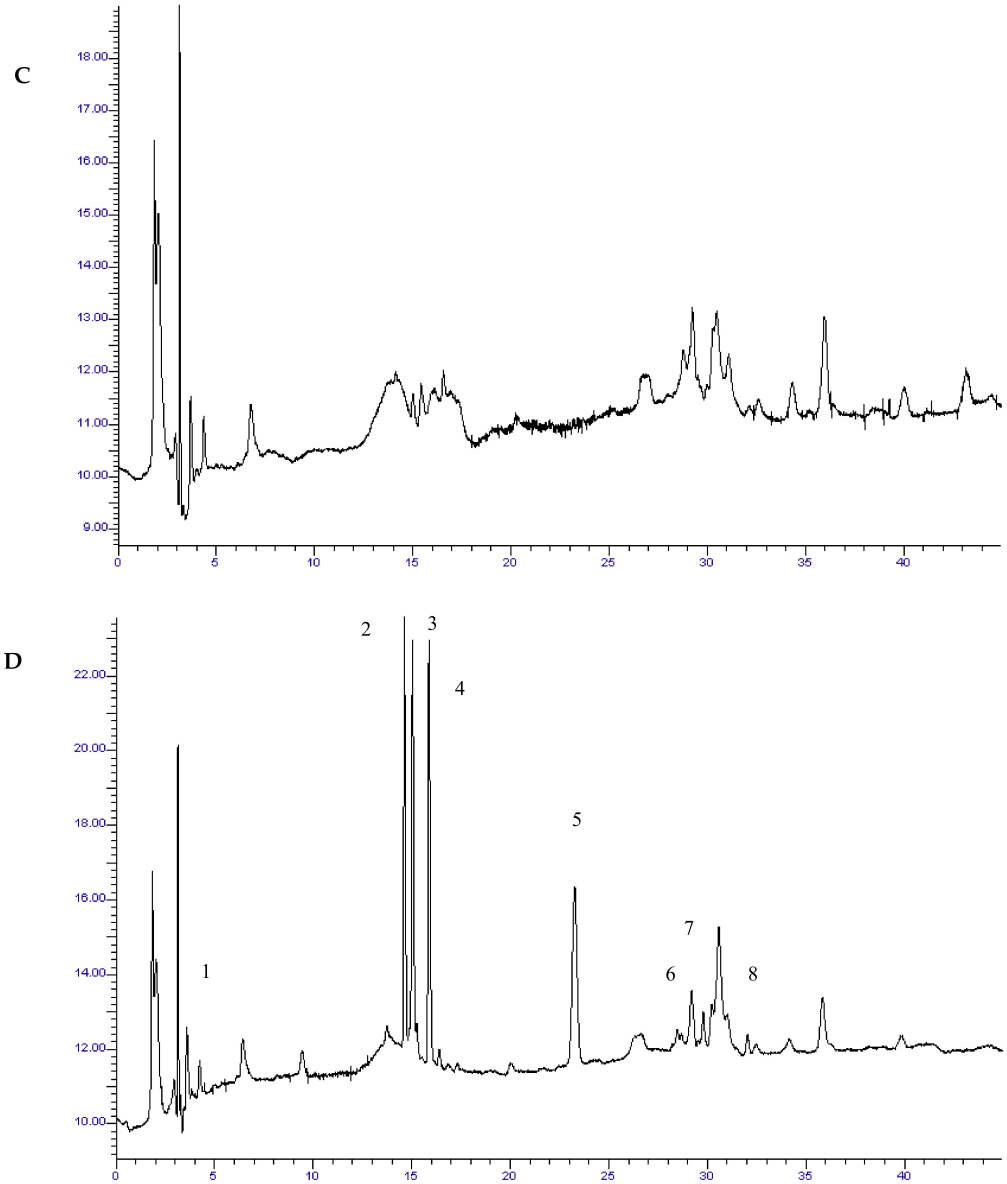

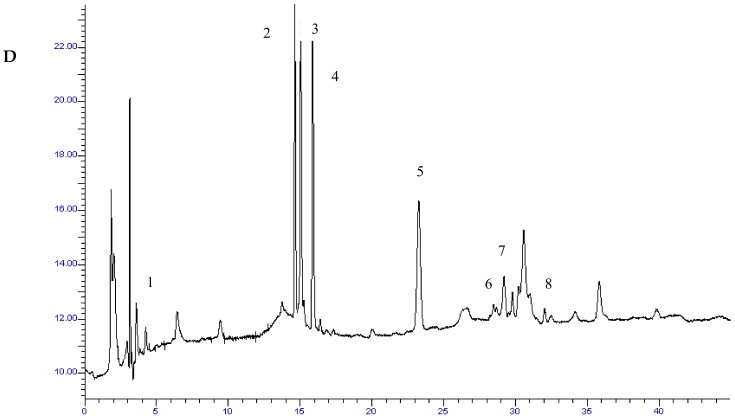
Chromatogram of blank milk sample at 240 nm. (**B**) Chromatogram of spiked milk sample with standard solution of target analytes (250 μg kg^−1^) at 240 nm. (**C**) Chromatogram of blank milk sample at 265 nm. (**D**) Chromatogram of spiked milk sample with standard solution of target analytes (250 μg kg^−1^) at 265 nm. Peaks: 1. AMO: 3.2 min, 2. CFZ: 14.8 min, 3. CFN: 15.2 min, 4. CFP: 15.8 min, 5. CFU: 22.5 min, 6. OXA: 29.8 min, 7. CAP: 30.4 min, 8. CLO: 32.3 min.

Preconditioning of Plexa sorbent was found to improve its performance. The contents of a QuEChERS tube were equally divided into two aliquots and blended with the Plexa sorbent. Moreover, sonication provided an efficient contact between the solid and the extractant, thus resulting in higher recovery rates of the target analytes. Absolute recoveries after ultrasound assisted MSPD ranged from 75–94% for all compounds. All examined analytes were well resolved from complex milk matrix. Typical chromatograms of blank and spiked milk samples after MSPD are illustrated in Figure 1.

### 3.3. HPLC-DAD Method Validation

The developed HPLC-DAD method was validated in terms of sensitivity, linearity, decision limit (*CCa*) and detection capability (*CCb*), trueness and accuracy, according to European Decision 2002/657/EU performance criteria. Samples of raw milk, which were analyzed and found not to contain detectable residues of the analytes, were used as negative controls (blank samples). The results of validation were calculated for individual substances.

Calibration curves were constructed using fortified milk samples after MSPD, and good correlation coefficients of between 0.989 and 0.999 were achieved over the examined range (20–2000 μg kg^−1^). All observed LODs were lower than the respective MRL values for the target analytes [27] (Table 3).

The precision of the method based on within-day repeatability was assessed by replicate measurements (*n* = 3) from three spiked milk samples at concentration levels of 0.5 × MRL, MRL and 1.5 × MRL. Relative recovery rates from the spiked samples were determined at the same concentrations by comparing the peak area for extracted β-lactams and chloramphenicol and the values derived from the respective calibration curve.

The between-day precision of the method was established using milk samples at the same concentration range as above. A triplicate determination of each concentration was conducted during routine operation of the system over a period of three consecutive days. RSD values for all the examined antimicrobial agents were lower than 10.7%, while mean apparent recovery rates for all target compounds ranged from 81.8% to 116.9% (Table 3).

The *CCa* values revealed after spiking 20 blank milk samples at MRL and *CCb* values by analyzing 20 blank spiked samples at the corresponding *CCa* level for each analyte are presented in Table 3, together with all values derived from validation procedure for examined parameters.

### 3.4. Microbial Inhibitor Test Kits’ Comparative Evaluation

Regarding β-lactams, all kits presented fairly good results either being interpreted photometrically or visually, as they were all able to detect the cephalosporins and penicillins at concentrations below the MRL values, except for four antibiotics. More precisely, kits “B” and “C” were able to detect the ampicillin residues at concentrations lower than the MRL values (2 μg kg^−1^ and 3 μg kg^−1^), whereas kit “A” was able to detect the antibiotic residues only visually at the concentration of 3 μg kg^−1^. However, kit “D” was not able to detect the ampicillin residues at none of the aforementioned concentrations. As far as ceftiofur is concerned, all kits could detect the antibiotic residues at the concentration of 75 μg kg^−1^, but only kits “A” and “B” were able to detect the ceftiofur residues at the lowest concentration (50 μg kg^−1^). Regarding cloxacillin and ampicillin, only kit “C” was able to detect the antibiotics residues at the concentrations of 15 μg kg^−1^ and 3 μg kg^−1^ respectively, either visually or photometrically, whereas none of the kits could detect the above residues at the lowest concentrations (5 μg kg^−1^ and 2 μg kg^−1^ respectively). However, even in those cases, all the test kits were able to detect the antibiotic residues at the MRL values, either visually or photometrically.

In the present study, milk samples were spiked with chloramphenicol at six concentrations: 5, 10, 15, 20, 25 and 30 μg kg^−1^. However, none of the commercial microbial inhibitor test kits were able to detect any of the spiked concentrations of chloramphenicol in milk. This fact comes in correlation with the ranges reported in the test kits’ sensitivity charts and is confirmed by the results of previous studies [28] as *Bacillus stearothermopilus* is considered to have sufficient sensitivity to the group of β-lactams, unlike other antibiotics [22,29].

### 3.5. Quality Control of Raw Milk Samples by Means of a Comparative Study Among the HPLC-DAD Method Developed and Microbial Inhibition Assays

Several samples of cow’s and sheep’s raw milk were provided to the laboratory in order to be examined for the presence of β-lactams and chloramphenicol residues. The samples were collected at individual cattle and sheep milk-producing facilities in three regions in the west, north and central Greece and were initially analyzed by the local Milk Quality Control Laboratories of Hellenic Agricultural Organization—“DEMETER”—using screening methods.

Firstly, the samples were analyzed by the microbial inhibition assays in duplicate according to the manufacturers’ guidance and the results were interpreted visually and photometrically. During the analyses, the milk samples were examined for any inappropriate consistency that could interfere with the kits’ sensitivity. The results of the analysis of the tests “A”, “B”, “C” and “D” are presented in Table 4.

From the analysis of negative milk samples, the microbial inhibitor test kits showed similar results to those obtained by the milk quality control laboratories of Hellenic Agricultural Organization—“DEMETER”. During the monitoring of the positive milk samples, the test kits “A”, “B” and “D” detected antibiotic residues in 10 out of 14 samples, whereas kit “C” detected antibiotic residues in 9 out of 14 milk samples. The spoilage of the milk samples, which was observed prior to analyses due to improper storage and shipping, probably increased the possibility of inactivation of the antibiotics, a fact that lead to false results due to loss of kits’ sensitivity. These observations are in correlation with the findings of previous studies [30].

Subsequently, milk samples that were found to be positive and those which were equivocal negative according to the results of the analysis with microbial inhibition assays, were pre-treated with the MSPD protocol described in Section 2.5 and then analyzed by the HPLC-DAD method developed herein. The results obtained confirmed the presence of antibiotic residues which in most cases surpassed the permissible limits. Experimental results are presented in Table 4.

According to the results obtained, 11 samples were found to be positive for β-lactams with concentrations above the permissible limits, which is in correlation with previous studies conducted in Italy [31] and Turkey [32], which have shown the presence of β-lactams residues at concentrations higher than the MRLs. Additionally, the results of the milk samples’ analytical determination in Northern Italy demonstrated that β-lactams are the more frequent antibiotic residues found in milk, a fact which enhances the results of our study, as most of the antibiotic residues found belonged to the group of β-lactams.

Concerning chloramphenicol residues, our results indicated its presence only in two cow’s milk samples. These results are similar to those obtained in previous studies performed in Croatia [33] and Turkey [34,35], which detected chloramphenicol residues in some of their analyzed milk samples. 

## 4. Conclusions

During their comparative evaluation, all microbial inhibitor test kits presented fairly good sensitivity at detecting β-lactams residues but were not successful in detecting chloramphenicol residues in milk. This fact is in agreement with the findings of previous studies, as *Bacillus stearothermopilus* is considered to have sufficient sensitivity to the group of β-lactams, unlike other antibiotics.

Taking into account the milk samples’ quality control, microbial inhibition assays could be successfully applied in the analysis of different types of milk. However, regarding the analysis of the positive milk samples using MIAs, the fact that some results of this study were in contrast to the results reported by the milk quality control laboratories of Hellenic Agricultural Organization—“DEMETER” and the HPLC method could be attributed to milk samples’ spoilage, which was observed prior to the analyses and was due to inappropriate storage and shipping, as described before.

The ultrasound-assisted MSPD method developed herein was successfully applied to the multi-residue analysis of β-lactams and chloramphenicol residues in milk by HPLC-diode array detection. The sensitivity of this method allowed quantifying all antimicrobials in milk samples, whereas milk’s spoilage does not affect the effectiveness of the analysis. 

## Figures and Tables

**Table 1:** Detection limits for selected antibiotics for the examined test kits.

Antibiotic Name	Spiked Antibiotic Concentrations (μg kg^−1^)						MRL (μg kg^−1^)	Comments
CEPHAZOLIN	25	40	50	75	100	125	50	Photometrical and visual detection
“A”	+	+	+	+	+	+		
“B”	+	+	+	+	+	+		
“C”	+	+	+	+	−	−		
“D”	+	+	+	+	+	−		
CEPHALONIUM	10	15	20	30	40	50	20	Photometrical and visual detection
“A”	−	+	+	+	+	+		
“B”	+	+	+	+	+	+		
“C”	+	+	+	+	+	+		
“D”	+	+	+	+	+	+		
CEPHAPIRIN	30	45	60	90	120	150	60	Photometrical and visual detection
“A”	+	+	+	+	+	+		
“B”	+	+	+	+	+	+		
“C”	+	+	+	+	+	+		
“D”	+	+	+	+	+	+		
CEFTIOFUR	50	75	100	150	200	250	100	Photometrical and visual detection
“A”	+	+	+	+	+	+		
“B”	+	+	+	+	+	+		
“C”	−	+	+	+	+	+		
“D”	−	+	+	+	+	+		
CLOXACILLIN	5	15	20	30	45	60	20	Photometrical and visual detection
“A”	−	−	+	+	+	+		
“B”	−	−	+	+	+	+		
“C”	−	+	+	+	+	+		
“D”	−	−	+	+	+	+		
OXACILLIN	10	20	30	40	60	75	30	Photometrical and visual detection
“A”	+	+	+	+	+	+		
“B”	+	+	+	+	+	+		
“C”	+	+	+	+	+	+		
“D”	+	+	+	+	+	+		
AMPICILLIN	2	3	4	6	8	10	4	
“A”	−	− *	+	+	+	+		* No photometrical detection at 3 μg kg^−1^
“B”	+	+	+	+	+	+		Photometrical and visual detection
“C”	+ **	+	+	+	+	+		** No visual detection at 2 μg kg^−1^
“D”	−	−	+	+	+	−		Photometrical and visual detection
AMOXICILLIN	2	3	4	6	8	10	4	
“A”	−	−	+	+	+	+		Photometrical and visual detection
“B”	−	−	+	+	+	+		Photometrical and visual detection
“C”	−	+	+	+	+	+		Photometrical and visual detection
“D”	−	− ***	+	+	+	+		*** No visual detection at 3 μg kg^−1^
CHLORAMPHENICOL	5	10	15	20	25	30	Banned	Photometrical and visual detection
“A”	−	−	−	−	−	−		
“B”	−	−	−	−	−	−		
“C”	−	−	−	−	−	−		
“D”	−	−	−	−	−	−		

*: Kit “A” was the only kit which could not detect ampicillin at the concentration of 3 μg kg^−1^ photometrically in comparison with the other kits. **: Kit “C” was the only kit which could not detect ampicillin at the concentration of 2 μg kg^−1^ visually in comparison with the other kits. ***: Kit “D” was the only kit which could not detect amoxicillin at the concentration of 3 μg kg^−1^ visually in comparison with the other kits.

**Table 2:** Optimization of Matrix Solid-Phase Dispersion (MSPD) procedure.

Trial	Sorbent	Elution	Washing Step	Observations
1	Plexa	2 mL MeOH	none	Not sufficient matrix cleanup.
2	Plexa + 125 mg QuECheRS	2 mL MeOH	5 mL H_2_O (5% acetone)	Target analytes are not well resolved from complex milk matrix.
3	Plexa + 125 mg QuECheRS	2 mL MeOH	2 × 5 mL H_2_O (5% acetone) successively	Target analytes are not well resolved from complex milk matrix. The amount of acetone added reduces absolute recoveries.
4	Plexa + 250 mg QuECheRS	2 mL MeOH	2 × 5 mL H_2_O (1% acetone) successively	The amount of QuECheRS sorbent interferes in the sufficient elution of target analytes.
**5**	**Plexa + 125 mg QuECheRS**	**2 mL MeOH**	**2 × 5 mL H_2_O (1% acetone) successively**	**All analytes are well resolved from milk matrix. Absolute recoveries ranged from 75–94%.**

The bold presents that which protocol was selected.

**Table 3:** Validation parameters for the determination of β-lactams and chloramphenicol in milk.

	Validation Parameters/Respective Values Obtained								
Compounds	Linearity *R*^2^	Slope	Intercept	MRL (μg/kg)	LOD (S/N = 3.3) (μg/kg)	Intra-Assay Recovery (*n* = 9) RSD%	Inter-Assay Recovery (*n* = 9) RSD%	*CCa* (μg/kg)	*CCb* (μg/kg)
AMO	0.998	40.18	67.27	4	1	97.5–106.0% 3.4%	109.5–110.9% 5.5%	4.2	5.2
CFZ	0.994	101.6	1070.0	50	7	81.8–102.1% 7.6%	95.6–102.1% 9.3%	51.2	53.7
CFN	0.998	82.89	624.3	20	4	90.5–95.7% 10.7%	98.5–106.1% 8.0%	22.1	24.2
CFP	0.996	39.53	941.8	60	7	107.5–11.7% 3.4%	105.8–113.2% 1.7%	61.6	67.6
CFU	0.998	76.83	662.0	100	7	87.5–101.8% 5.7%	89.9–102.5% 3.2%	105.3	110.7
OXA	0.997	105.6	615.0	30	4	91.5–100.5% 8.6%	95.4–101.4% 3.0%	32.6	34.7
CAP	0.999	85.05	1498.0	-	4	97.4–116.9% 4.1%	99.3–109.5% 3.0%	31.1	33.7
CLO	0.989	55.73	1604.0	30	4	86.8–97.5% 8.9%	96.9–100.9% 10.7%	31.8	36.1

**Table 4:** Results of the analysis of milk samples with microbial inhibition assays and HPLC-DAD method developed.

Geographical Region	Milk Type	Sample Code No.	Microbial Inhibition Assays				HPLC-DAD Antimicrobials Detected
			A”	“B”	“C”	“D”	
1	Cow’s milk	5L	+	+	+	+	CFU
		8L	+	+	+	+	CAP, OXA
		11L	+	+	+	+	CFN, CFP
2	Sheep’s milk	8G	−	−	−	−	CLO
		10G	−	−	−	−	CLO
		1P	+	+	+	+	CFZ
3	Cow’s milk	2P	+	+	+	+	CFN
		3P	−	−	−	−	CFP
		5P	+	+	+	+	OXA, CAP, CFZ, CFP
		6P	−	−	−	−	AMO, CFN
		7P	+	+	+	+	OXA, CFP

The initial letters L, G and P refer to the milk samples’ origin.
